# “Taking your place at the table”: an autoethnographic study of chaplains’ participation on an interdisciplinary research team

**DOI:** 10.1186/s12904-015-0006-2

**Published:** 2015-05-02

**Authors:** Allison Kestenbaum, Jennifer James, Stefana Morgan, Michele Shields, Will Hocker, Michael Rabow, Laura B Dunn

**Affiliations:** Center for Pastoral Education, Jewish Theological Seminary, 3080 Broadway, New York, NY USA; Department of Social and Behavioral Sciences, University of California, San Francisco, USA; Department of Psychiatry, University of California, San Francisco, USA; Spiritual Care Services Department, University of California San Francisco Medical Center and UCSF Benioff Children’s Hospital, San Francisco, USA; Department of Medicine, University of California San Francisco, San Francisco, USA; UCSF Helen Diller Family Comprehensive Cancer Center, San Francisco, USA

**Keywords:** Chaplains, Spiritual care, Palliative care, Qualitative research, Ethnography, Interdisciplinary teams

## Abstract

**Background:**

There are many potential benefits to chaplaincy in transforming into a “research-informed” profession. However little is known or has been documented about the roles of chaplains on research teams and as researchers or about the effects of research engagement on chaplains themselves. This report describes the experience and impact of three chaplains, as well as tensions and challenges that arose, on one particular interdisciplinary team researching a spiritual assessment model in palliative care. Transcripts of our research team meetings, which included the three active chaplain researchers, as well as reflections of all the members of the research team provide the data for this descriptive, qualitative, autoethnographic analysis.

**Methods:**

This autoethnographic project evolved from the parent study, entitled “Spiritual Assessment Intervention Model (AIM) in Outpatient Palliative Care Patients with Advanced Cancer.” This project focused on the use of a well-developed model of spiritual care, the Spiritual Assessment and Intervention Model (Spiritual AIM). Transcripts of nine weekly team meetings for the parent study were reviewed. These parent study team meetings were attended by various disciplines and included open dialogue and intensive questions from non-chaplain team members to chaplains about their practices and Spiritual AIM. Individual notes (from reflexive memoing) and other reflections of team members were also reviewed for this report. The primary methodological framework for this paper, autoethnography, was not only used to describe the work of chaplains as researchers, but also to reflect on the process of researcher identity formation and offer personal insights regarding the challenges accompanying this process.

**Results:**

Three major themes emerged from the autoethnographic analytic process: 1) chaplains’ unique contributions to the research team; 2) the interplay between the chaplains’ active research role and their work identities; and 3) tensions and challenges in being part of an interdisciplinary research team.

**Conclusions:**

Describing the contributions and challenges of one interdisciplinary research team that included chaplains may help inform chaplains about the experience of participating in research. As an autoethnographic study, this work is not meant to offer generalizable results about all chaplains’ experiences on research teams. Research teams that are interdisciplinary may mirror the richness and efficacy of clinical interdisciplinary teams. Further work is needed to better characterize both the promise and pitfalls of chaplains’ participation on research teams.

## Background

Modern health care chaplaincy is transforming into a “research-informed profession” [1]. Research will advance the field as an evidence-based profession, establish and disseminate evidence of chaplains’ value for diverse health care populations and settings, and point toward novel research questions relevant to chaplaincy and spiritual care that should be examined [2-7]. However, little is known about chaplains’ roles as researchers, how chaplains interact with other members of the research team, or the effects of research engagement on the research process or on chaplains themselves.

Autoethnography is a type of qualitative research method that “seeks to describe and systematically analyze (*graphy*) personal experience (*auto*) in order to understand cultural experience (*ethno*)” [8]. During the course of an interdisciplinary study on spiritual assessment and intervention, the three chaplains on the research team spontaneously reflected on how participating in the research project was affecting them personally and professionally. In order to capture these reflections, the team audio-recorded the weekly team meetings. The transcripts of these team meetings, as well as our individual reflections on the team’s process, provide the data for this descriptive, qualitative, autoethnographic analysis.

This analysis aims to enhance understanding of the experience of chaplains as active members of research teams, through an autoethnographic description of one interdisciplinary team that conducted a mixed-methods study of a specific spiritual care assessment and intervention model [9]. We describe effects of research team participation on the chaplains, as captured in their own words and reflections, and in interactions with other team members. We also explore chaplains’ contributions to the interdisciplinary research process, as well as the tensions and challenges that arose for chaplains during the parent research project.

## Methods

### Context: the Spiritual AIM research study

This autoethnographic project evolved from a parent study, entitled “Spiritual Assessment Intervention Model (AIM) in Outpatient Palliative Care Patients with Advanced Cancer” which focused on the use of a well-developed model of spiritual care, the Spiritual Assessment and Intervention Model (Spiritual AIM) [9]. This model is described in detail elsewhere [9]. The catalyst for the parent study was an award from the HealthCare Chaplaincy and The John Templeton Foundation for rigorous research projects that not only identified and explored hypotheses regarding chaplains’ contributions to palliative care, but also incorporated chaplains into the study in meaningful ways [10]. In addition to being mentored in all aspects of research by the PI, at least one “chaplain researcher” on each team attended four symposia as part of the project with other grantees and participated in seminars and consultation with experts in quantitative, qualitative and spiritual care research. The title of this paper, “taking your place at the table” was inspired by and echoes statements made by symposium faculty throughout the 18-month study encouraging those assembled to “stake a claim” in these early stages of chaplaincy research and interdisciplinary teams actively including chaplains.

**Figure 1:**
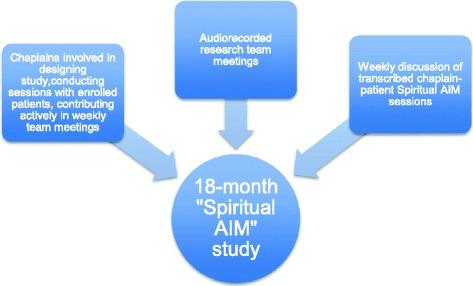
**Components of parent Spiritual AIM study.**

The goals of the Spiritual AIM study were to provide preliminary, mixed-methods data regarding the process, content, and impact of Spiritual AIM in the outpatient palliative care setting (see Figure 1 for components of Spiritual AIM study). Thirty-one patients with advanced cancer were recruited from an outpatient palliative care service at an academic, urban comprehensive cancer center. All patients were receiving concurrent oncologic and palliative care. The inclusion criteria were an advanced cancer diagnosis and willingness to speak with a chaplain for three one-on-one visits, either in person or by telephone. A core component of the Spiritual AIM research process involved weekly interdisciplinary team meetings. (Table 1). The main findings of the Spiritual AIM parent study will be presented in a separate report.

**Table 1:** **Spiritual AIM research team composition**

**Role on team**	**Specialty**	**Research experience**	**Prior experience with using Spiritual AIM**
Principal Investigator (PI)	Professor of Psychiatry and Director of Psycho-Oncology at the Cancer Center. Board Certified in Psychiatry and Geriatric Psychiatry	>14 years of experience as a clinical researcher	None
Chaplain 1	Director of Spiritual Care Services. Ordained United Methodist minister, Board Certified Chaplain (BCC), Association of Clinical Pastoral Education Supervisor	None	Developed the Spiritual Assessment and Intervention Model. Has been teaching the model to chaplain trainees for over 20 years
Chaplain 2	Lead chaplain-investigator. Board Certified Jewish Chaplain (BCC) and Association of Clinical Pastoral Education Supervisor	Master’s level training that included social science research	Has been utilizing Spiritual AIM for over 8 years
Chaplain 3	BCC eligible ordained Episcopal priest. Previously worked as a master’s level therapist	Some research background in previous career	Has worked with the model for several years. Trained in Spiritual AIM by the other 2 chaplain research team members for the preceding 3 years
MD Co-Investigator	Professor of Clinical Medicine and Director of the Symptom Management Service at the Cancer Center. Board Certified in Hospice and Palliative Medicine	Experienced in clinical research (including in spirituality and palliative care)	None
Research Coordinator	PhD candidate in sociology with a background in policy and in social work	Extensive experience with qualitative research and was the member of the core team most involved in day-to-day data management	None
Other	Additional individuals were sometimes present as well (e.g., medical students participating in research electives, chaplain trainees interested in gaining exposure to research)	Minimal	Chaplain interns: some; Medical students: none

### Analytic method: autoethnography

The primary methodological framework for the analysis of the team process was autoethnography [8]. Carolyn Ellis describes the field of autoethnography as an approach to research and writing that “seeks to describe and systematically analyze (*graphy*) personal experience (*auto*) in order to understand cultural experience (*ethno*)” [8]. When researchers endeavor to do ethnography, they retrospectively describe epiphanies that are derived from being part of a culture or having a particular cultural identity.

Our research process and team dynamic were unique in that chaplains were active participants on the research team as well as subjects of the research study on Spiritual AIM. In addition, team meetings served as both a tool for analysis in our larger project as well as the site of inquiry and analysis for work aimed at better understanding the chaplain as researcher. Thus, we sought to use autoethnography not only to describe the work of chaplains as researchers but also to reflect on the process of researcher identity formation and to offer personal insights regarding the challenges accompanying this process.

This report will be part reflexive ethnography as we document the way the researcher changed as a result of engaging this research [11]. Additionally, we will use co-constructed narratives to describe how our team collaboratively approached challenges in research [11]. This work is based on the experiences of our research team and, primarily, on the work done in parent study team meetings. In addition to participant observation in these sessions, each session was audiorecorded and analyzed both collectively and independently by several members of the research team. All research participants and research team members provided written informed consent for this study. The Spiritual AIM study was reviewed and approved by the UCSF Committee on Human Research.

For this paper, transcripts of nine parent study team meetings were reviewed, as were individual notes (typed during the team meetings by the research coordinator, PI and lead chaplain researcher) from team meetings. We began by open coding in a group setting with 3 different team members reading transcripts and noting themes that emerged. We then coded each transcript based on the set of six major themes identified. After coding was completed we pulled the coded section of data and compared it to our notes from team meetings. Team members interviewed each other about their memories from our team meetings focusing on the themes that emerged in the coding. Individual team members were also asked to memo throughout the team process on their observations, feelings, opinions and revelations. “Reflexive memoing,” a common qualitative method used to address individual and collective bias in the research process, facilitates the examination and evaluation of ongoing interactions with research participants and team members [12]. These memos were analyzed using the same coding framework as the transcripts of team meetings to develop a fuller picture of the impressions of team members.

## Results

Three major themes emerged: (1) chaplains’ unique contributions to the research team; (2) the interplay between the chaplains’ active research role and their work identities; and (3) tensions and challenges related to participation on an interdisciplinary research team. Each major theme, as well as subthemes, are described below.

### (1) Contributions of chaplains to the research team

Team meetings centered around an emphasis on open dialogue. Each member of the team was encouraged to bring ideas and questions to the table. The non-chaplain team members often asked extensive questions to better understand chaplaincy, spiritual care—in particular, Spiritual AIM and its use by the chaplains—and how the chaplains approached and conceptualized their patients and their process. Transcripts of the parent study team meetings revealed ways in which the chaplains played crucial roles as educators of the other research team members.

First, chaplains spent time in meetings articulating basic concepts of pastoral care and highlighting—as well as demystifying—theologies to explain observed phenomena and patient behavior. For instance, when one chaplain described a patient’s theology as “childlike,” another chaplain helped the team understand the broader context of this comment by citing a specific faith development theory that supported and expanded upon the first chaplain’s description, simultaneously educating the team about faith development [13]. What at first seemed to the non-chaplain team members as a pejorative term was reframed as an important clinical observation for chaplain intervention, without a negative connotation or value judgment attached.

Second, the chaplains found that they needed to further describe or “translate” their professional jargon for the non-chaplain team members. This often led to a member of the team from another discipline attempting to restate chaplaincy terminology in their own words. As in the following example, this led to discussions about whether the terms from the two different disciplines were in fact equivalent, or rather if they were similar but carried particular, discipline-specific nuances.

Chaplain 1: And the “meaning and direction” people don’t know who to blame. They just kind of muse on the question.

PI: So what are they though? What are they in psychiatric terms? I don't know what we would --

Chaplain 2: I have a “meaning and direction” patient. The research coordinator confirmed it last week when she said that when she read the transcript and she was like, “What is going on here? What is she talking about?” [Laughter]

PI: Oh, histrionic! That’s what popped into my head.

Chaplain 1: Now we have some psychological terms to pass around.

Such discussions contributed, unexpectedly, to the richness of the interprofessional dialogue and helped deepen the non-chaplains’ understanding of chaplaincy and spiritual care. It also improved the chaplains’ understanding of psychology and psychiatry terms and diagnostic categories. This is addressed in more detail in the next section. With a greater understanding and evidence base, chaplains were better able to distinguish the model from psychosocial tools. “In dialogue with representatives of other disciplines on the team, I could see how our model provided a different ‘lens’ through which to view the patient from that of a psychiatrist or social worker, for example,” said one chaplain.

In another example, the PI reflected publicly—while introducing the team’s work at a national conference on health care chaplaincy research—on the personal impact of working with the chaplains on the research project:

*“As a psychiatrist, as an academic and skeptical researcher, this project represents a journey—and, to put it simply, a revelation. I went from knowing essentially nothing about what chaplains do, to curious about what chaplains do, to fascinated by what chaplains do, to committed to bringing empirical methods to understanding what chaplains do, to convinced that chaplains can and do bring unique, crucial, and potent skills to the care of our patients, and finally to passionate about telling everyone else what chaplains do, how they do it, and why it is so important.”*

Third, the chaplains educated other research team members about less-understood aspects of chaplains’ professional practice. In the following example, as the team reviewed a session transcript, the chaplain explained to the team that her goal in the session was to invite the patient to give voice to her anger toward God. The patient previously had expressed her reluctance or inability to do so in church, and the chaplain assessed that expressing anger might spark further spiritual healing.

Chaplain 1: Yeah, I wanted to give her permission to air that grievance because there is a biblical tradition for speaking back and saying, “Wait a minute. What you like and what’s going on with you, God, is not what I like…”

PI: …it occurs to me that what you’re saying is not something that an uninformed person like myself would know, would have any idea is part of what chaplains do, namely give people permission to have this kind of --

Chaplain 1: Dialogue with God?

PI: Yeah…I think the ignorant view is that…you come in and pray with people or encourage people to…be at peace with God.

Chaplain 1: Right. And her own faith gives her options to have a broader expression – a broader emotional expression -- which is what she’s already saying here to God. “What you think is best is not maybe what I think is best for me, God.” And that is a genuine prayer of her heart and she’s already said it and she’s laughing…

### (2) Interplay between the chaplains’ active research role and work identities

Participating actively on the research team had several effects on the identity, theory, practice, and confidence of the chaplains. First, the chaplains asked questions about and therefore learned from other team members about research methods, protocols, ethics and etiquette. For example, the lead chaplain investigator initially worried that research might reveal gaps that would undermine the profession, but quickly learned from the other team members and by participating in the project itself that research instead uncovered many strengths as well as opportunities for further exploration. Chaplains’ empirical inquiries and hypotheses were validated as legitimate research questions by the other members of the team. Research became less threatening and became a means for chaplains to ask questions that bring to light and build professional strengths. During one team meeting the lead chaplain reflected, “One of the things that I’ve loved so much about the research is that it gives me this security that there can be these gaps and there can be these places where it didn't work the way we thought it would. But that creates intrigue. That’s where the grist is. It’s a little bit of a different stance for us.” Rather than research being in opposition to spiritual work, the chaplain found that research could help both to strengthen the work of chaplains as well as teach others about the work that chaplains do.

The other chaplains agreed that, particularly in a field often beset by concerns about “tangibility” or “reducibility” of chaplains’ work with patients, participating in an empirical study brought further credence and accountability to their clinical work. The PI and Chaplain 3 (who had practiced as a therapist prior to becoming a chaplain) often compared the current state of chaplaincy research to that of psychotherapy research several decades ago. This perspective demystified the research process and gave chaplains a far-reaching perspective into the future of their profession.

Second, all chaplains felt visible, encouraged and welcomed as they took their place at the research table. Furthermore, the skills they learned enhanced their curiosity and confidence to take on new leadership roles within this research project. In fact, soon after the conclusion of the Spiritual AIM study, the lead chaplain initiated a new qualitative research project in a new setting. The chaplain reflected, “I have learned to be very disciplined about articulating the problem and research gap around which the project is designed. I can see how tempted I have been to bite off more than I can chew…with research. For example, from participating in the Spiritual AIM study, I came to understand how important and legitimate qualitative research is. I don’t need to use [quantitative] measures in my new study just for the sake of doing so.”

Third, another chaplain recognized that integrating research methods into chaplaincy as a field might acknowledge, and perhaps eventually begin to ease, a tense relationship between religion and science, particularly in physical and mental health care: “I think there’s a distrust of science. Science and religion have a very tenuous relationship.” Another chaplain, who directed Spiritual Care Services at the medical center, stated, “What I’ve found is a level of respect or curiosity, but not hostility, about this research. I’m learning a language that helps me communicate better with people who are more scientifically or research-oriented, the academic medicine research community.”

Through team participation one chaplain came to understand that there is a wide range of roles for chaplains on research teams. Some of these roles require a modest time commitment and may include little to no expectation for chaplains to write. The chaplains’ presence on teams working on spiritual care-related research is critical. For example, for our project, the participation of two very experienced chaplains was vital because of their insight into patients’ spiritual background and religious needs. In addition, their expertise, education and intuition proved indispensible when the research team developed the coding scheme that identified and defined spiritual needs.

Fourth, as a result of their research work, chaplains’ clinical work was transformed. Chaplains reported an intention to use the Spiritual AIM model more thoughtfully in working with patients. The two chaplains who were newer to the Spiritual AIM model believed that their engagement with the Spiritual AIM study led to them conducting more effective assessments and interventions. Here, the chaplain who was least experienced in using Spiritual AIM (Chaplain 3) engages in discussion with the more experienced chaplains and the other team members regarding the spiritual assessment of one of his patients.

PI: Let me ask you. When you were in your first meeting with him, were you starting to formulate an assessment? How does it work for you?

Chaplain 3: Early on, I began to think “Self-Worth”….I don’t know, let me go way back…

Chaplain 2: It’s so funny because I projected onto you a Meaning and Direction intervention in as early as line 58… it was a conversation about wondering. [Chaplain 3] Did some wondering with him, alongside him. Which is a [“meaning and direction”] intervention.

Chaplain 3: I don’t know what I was thinking at the moment but it could have been me sort of, hearing “Meaning and Direction” at that moment. I honestly -- I don’t know, but I see what you’re saying.

Chaplain 2: Yeah. Well, you could argue that it’s [“Self worth and Belonging to] Community” also. You could argue it both ways.

Chaplain 3: I guess what I want to say is: So let’s just say if he were “Self-Worth”, clearly, three sessions, we did not get very far, you know what I mean? He clearly feels connected to me. I can tell we really like one another. I have such affection for this guy, I can’t tell you. And I sort of felt the mutuality in that. But clearly, we didn’t -- I feel like we didn’t get very far.

PI: Do you think you would feel that [you didn’t get very far] with someone who was more “Meaning and Direction”?

Chaplain 1: Yeah, ‘cause if you felt like you were spinning your wheels….

Chaplain 3: Right. That’s a classic “Meaning and Direction” experience.

Chaplain 1: That’s a classic response to kind of “Meaning and Direction”. It’s kind of like you’re going around in circles with him, and you didn’t go very far. You gave him plenty of affirmation and that didn’t work, right?

Chaplain 3: Right.

Chaplain 1: So that kind of eliminates the “Self-Worth thing”, right?

The passage above demonstrates the sometimes challenging, limit-pushing, almost Socratic approach that emerged in our discourse while analyzing the parent study transcripts. The model itself was “in play” in this passage, as the team worked through their impressions of the patient and asked Chaplain 3 about his reactions to the patient. These interactions between chaplains of different experience levels allowed non-chaplain team members to learn not only about the practical application of the model, but also to peer into the chaplains’ internal processes in using the model. This process of “thinking aloud” was a powerful instrument. It served to educate the non-chaplain team members about the chaplains’ work, and demystified the research process itself for the chaplains, as the non-chaplain team members asked questions and expressed genuine curiosity as part of the research process itself.

Fifth, chaplains whose other duties included clinical pastoral education supervision also reported an enhanced ability to articulate the model to chaplain trainees, who in turn expressed more receptivity and comprehension. Furthermore, the chaplain educators themselves found a way to balance their focus on evaluation with the scientific outlook of research. For example, through the coding, one chaplain noticed that “I was doing a lot less evaluating/critiquing, and could see the good quality of the work and the model came out more.” This was significant because much of chaplain training focuses on an “action/reflection” model of learning of Clinical Pastoral Education [14,15]. In this educational model, chaplains are encouraged to actively analyze and evaluate patient encounters on their own and seek out critique from peers and educators. While this action/reflection approach is important for the professional development of the chaplain, the research method is more dispassionate and does not seek to evaluate or improve the work of the chaplain.

Sixth, the research teamwork helped chaplains to reconcile their clinical and research perspectives. In one meeting, the chaplains discussed whether engaging in the research process—particularly coding their sessions with patients—felt in some way reductionistic. They concluded, however, that they felt their work with patients was well-represented by the codes, the essence of the work was captured, and they even felt their work enriched. The self-identified “newcomer” to the Spiritual AIM model, said that “a regular complaint we hear about model-making is that we lose the essence of the experience by making a model out of it, but here I feel like those distillations have actually, for me, helped me better articulate my understanding of what we’re doing. It also seems like our definitions are crisper and sharper.” Two of the chaplains reflected that the questions from the non-chaplains helped the chaplains refine their understanding of the model.

### (3) Tensions/challenges

There were also several tensions that that chaplains experienced on the research team. The biggest was feeling pulled between research, clinical and administrative responsibilities and not having as much time as they would like to devote to research related tasks and projects. One of the chaplains reflected on his feelings of not always being able to put as much time as he would have liked into the project.

*“[Chaplain 1] and I are working on how to take some things off my plate. When I said, Yes, yes, yes, pick me, I want to do this, I really didn’t have a sense of what my clinical job was going to be. [PI] asked me to clarify, and I said, I’m committed. I’ve been really disappointed by how little time I’ve been able to put into this. [PI] said, You’re fine, you’ve done a lot, you’ve done enough. But it’s not to my standard. Not that I don’t like this, but I can’t do this and my other job. And I love my other job. I’m just kind of disappointed. I feel like I’m tepidly here. I’m half here.”*

Other tensions centered around point of view, fidelity to Spiritual AIM and whether, how, and when to use “structure” or models at all. This is a common and basic question among chaplains in the field of spiritual care [16,17]. As the original author of the model said,

*“The beauty of Spiritual AIM is that it allows a chaplain to be in relationship to a patient, but does not dictate exactly how one must intervene at any particular moment. It is more art than science how one embodies a guide or a truth-teller or a valuer. While the chaplain accompanies the patient along a path to healing and wholeness, it doesn’t predict exactly how the patient will respond and allows great freedom to the chaplain to dance with the patient along the way.”*

A model needs to be used to evaluate the efficacy of chaplaincy, it was concluded.

Another challenge for our interdisciplinary team was negotiating interpersonal and power dynamics on the research team. The chaplains reflected that these power dynamics parallel those that occur in other interdisciplinary settings, such as clinical rounds in the hospital. They reflected that, in the research setting, assuring that each team member had an equal voice (e.g., asking each team member for their initial assessment after reading a session transcript) helped to “level the playing field.”

During the course of the research project, the chaplains also felt that they developed new skills in negotiating team dynamics. For example, one of the chaplains felt that she learned a great deal from one particularly uncomfortable disagreement that occurred in a team meeting. On this occasion, the two chaplains who were present agreed about their assessment of a patient's core spiritual need, but a non-chaplain team member disagreed quite assertively, causing some discomfort among the chaplains. One chaplain later reflected,

*“The thing that I learned from this disagreement, when there are polar opposites in terms of assessment in the room—between the chaplains on one side who know the model really well, and another person who doesn’t know the model quite so well, but feels really strongly that their assessment is correct—is to get curious. The idea is to not dig in, with my view of the assessment, but to get curious about what evidence do they see that informs their understanding. What is it that the chaplains see? And to have both sides talk about what evidence they see, and have them dialogue about it, so that both sides can learn, because maybe there’s something about the model, a gap, that needs to emerge, or maybe there’s something about the model that we need to learn. The big learning piece for me was that I didn’t have the skills in that moment to get curious. Now, recognizing that I was beginning to get defensive, I would say to myself, ‘get curious’.”*

She also reflected on the value of speaking with an outside consultant regarding challenges in team dynamics. In discussing this later with the PI, the PI concurred, stating that it was often very useful to consult with mentors or colleagues outside of one’s specific research team, particularly regarding challenging or difficult team dynamics.

During numerous team meetings and discussions, the chaplains agreed that a key quality they valued in non-chaplain collaborators was humility—openness to learning about spiritual care and the chaplains’ unique perspectives and experience. This was also part of the interdisciplinary team experience that others on the team noted and commented upon. As the research coordinator stated,

*“We had many productive disagreements and times of challenging each other in the team meetings. There were a few specific instances where it went really poorly. But one of the things I loved about working on this team is I could say to the chaplains, ‘I have no idea what you are talking about. This concept doesn’t make sense to me and have the chaplains explain it to me, and we would dialogue back and forth. I believe it helped us push the model in new ways and push how the model is explained. There are still patients that I disagree about the assessment that was made by the chaplain and it's been a good thing to talk about, Does that matter? Whose assessment is important? Why does the assessment matter? Those are the kinds of questions we can't get to unless we disagree sometimes. Those challenges and disagreements create a really beautiful space for more learning. You don't want to create a team where everyone agrees. You want to create a team where people can disagree really respectfully with each other.”*

Another dynamic that became visible in the context of the research teamwork was related to the varying levels of chaplaincy experience among the chaplain members of the team. There was tension in the chaplains’ need to separate their role of supervisor and educator from their role as researcher. The chaplains learned from each other, as occurred in the following exchange between the Director of Spiritual Services (Chaplain 1), and Lead Chaplain Researcher (Chaplain 2). Chaplain 2 was encouraged by the suggestion to approach interventions with greater confidence while Chaplain 1 was reminded to shift from a focus on critique of pastoral practice to that of clarifying Spiritual AIM through the rich chaplain-patient data.

Chaplain 1: I thought it was a really powerful image that you were offering her too, but it’s kind of taking away from saying it. “I just, I just, I just.” And it sounds like you were slightly hesitant in offering your image to her. So I’m wondering if you could use different language as you offer an image to a patient, like –

Chaplain 2: Can you help me understand how this relates to articulating Spiritual AIM?…I could see that if I’m wanting to embody the guide I want to do it with as much firmness, maybe not firmness, but as much clarity as possible.

The chaplains found great value in these opportunities to improve their practice by reading the transcripts of patient interactions. One of the chaplains reflected that reading through transcripts involved “drawing upon the same skills as reading verbatims, identifying whether the other chaplain was assessing the patient in a way that I would concur with or not. But I had to get out of the mindset of critiquing it as a CPE [Clinical Pastoral Education] supervisor and get to where I was discussing it with a more open mind.” (Verbatims, sometimes referred to as pastoral visitation reports, involve chaplain trainees recording, in writing, an encounter with a recipient of their pastoral care. The chaplain trainee answers a series of questions to aid in analyzing the material, to develop insights about the patient and effectiveness of their pastoral care, and to use these insights to modify future pastoral care.) The chaplains had to learn to shift their thinking away from being a chaplaincy supervisor to being a analytic researcher, while continuing to bring their unique skill set and perspectives to team meetings.

## Discussion

This autoethnographic study found that, as experts in providing clinical spiritual care, the three chaplains played crucial roles on our interdisciplinary research team. Non-chaplain team members pointed to ways that chaplains offered specific expertise, experience, and insights that enriched the research. Moreover, the chaplains themselves reported that they were enlarged as both researchers and clinicians as a consequence of their participation on the research team [18].

In our view, one of the most intriguing developments of the project was how over time, the non-chaplains came to recognize, understand and speak the language of the chaplains. This allowed the chaplain and non-chaplain team members to speak in a sort of shorthand that allowed for deeper understanding of the professional practice of chaplaincy and of its clinical and research implications. We found this to be especially remarkable because the chaplains and non-chaplains all acknowledged a lack of a common language about spiritual care on *clinical* interdisciplinary teams, that exists as part of a broader lack of clarity about chaplains’ role on care teams. Our team lamented the ways in which this lack impinges upon the ability of care teams to effectively address the spiritual dimensions in the care of patients. Traditionally, chaplains learn and translate the language of other health care disciplines in order to participate in the team and patient care. This study suggests that chaplains and non-chaplain researchers participating in research together may provide for a special environment that allows for a culture of curiosity. Perhaps the relative expansiveness of research compared to the busy clinical environment encourages chaplains and non-chaplains to work together to define some of the ambiguities of spiritual care provision which may lead to narrowing of clinical gaps.

In our study, the chaplains observed, and the non-chaplain team members confirmed, that during the course of the parent study this learning and translation became much more reciprocal. As part of an interprofessional research team, the chaplains expressed feeling that the project benefited from the deep commitment of the non-chaplain team members to understanding pastoral care theory and the distinct language of chaplaincy. Similar outcomes may be achieved by interdisciplinary research teams that are able to foster this same devotion to attaining deeper understanding.

However, this development of a “shared language” does not imply that the non-chaplains became as proficient in spiritual care as the chaplains. An important finding of our study is that the non-chaplains reported developing greater humility about their own lack of training in spiritual care, thanks to the a greater understanding of the depth and breadth of the chaplains’ training, experience, and expertise. The chaplains described this as an important validation of their professional role and unique contribution to patient care. Chaplains emerged in this study as spiritual care specialists on the parent study research team. Chaplains are experts about how to conduct spiritual assessments and diagnose how spiritual and religious beliefs and values impact patient care on interdisciplinary care teams [19]. Our study raises a hypothesis that this clinical role can be mirrored in research. Further authoethnographic studies where chaplains record their impressions are needed to provide additional data.

Our study is the first autoethnographic study of an interdisciplinary research team involving chaplains, and suggests that greater diversity of disciplines on a research team enriches the conversation. In the case of our study, we were able to use the expertise of each team member to look at our data from multiple perspectives and raise deeper and richer research questions. In our view, this deepened interprofessional awareness and appreciation was one of the most important and gratifying outcomes of this interdisciplinary project. Moreover, it is important to emphasize that interdisciplinary research teams can benefit from professional chaplains’ active engagement not only when spiritual care is the focus of the research, but—of particular relevance to palliative care and psychosocial research involving seriously ill patients—whenever patients are the focus of the research.

Limitations of this autoethnographic study should be noted. The composition of the research team (i.e. the number of chaplains and their prior relationships to each other, the disciplines of the non-chaplain team members) are unique to our team and would be difficult to replicate. Traditional social scientific research methods that rely on “large random samples of respondents” may indicate that this is a limit to generalizability to other research teams and clinical settings and suggest that all of interpretation of the data is subject to our own biases, based on our individual perspectives, training, experiences, and backgrounds [20-22]. However, our primary analytic method of autoethnography allows for these nuances. Authoethnography offers an understanding of generalizability that relies on *readers* of our findings to evaluate whether or not the conclusions are familiar and congruent with their own experiences [21,22].

Another unique component of our study was that it was conducted in the context of a parent study about spiritual assessment and intervention for palliative care outpatients. Leading hospice and palliative care organizations indicate that interdisciplinary palliative care clinical teams should include a chaplain because patients seen in palliative care typically have serious and often end-stage illness with the attendant spiritual concerns of meaning, loss and relationship [19]. Palliative care research must also address many of the complex issues now before chaplaincy research, including questions about the disciplines’ contributions to overall clinical benefit. Ultimately, palliative care is keenly invested in the developing scientific rigor of chaplaincy research as palliative care services nationwide strive to understand the unique contributions of each member of the team and must justify supporting a fully interdisciplinary team.

Future work examining chaplains as researchers should include evaluation of chaplains’ research experiences in diverse geographic and clinical settings, as well as with varied patient populations, in order to understand whether the experiences and perceptions described here exist outside of this team and outside of the palliative care setting. It would also be informative to examine chaplain experiences in research that is highly quantitatively focused, in contrast with the mixed-methods approach of our parent study. Moreover, as the parent study was a relatively short-term project (funding period of 18 months), there is a need to examine the longitudinal experience of chaplains and non-chaplain researchers who are engaged on longer-term studies, in which team processes, role, and dynamics are likely to evolve over time.

## Conclusions

Professional chaplains are trained to and have been caring for people of all or no faith for decades as well as attending to more universal spiritual needs. Their broad expertise, which is not limited to questions of faith or religion, has a place in research. Articulating how chaplains can participate in and contribute to research teams is critical to advancing chaplaincy research. Identifying the unique contributions of chaplains to the research team can encourage non-chaplain researchers to include chaplains on research teams. Moreover, helping chaplains see their value as members of the research team may help chaplains advocate for their inclusion on such teams. Furthermore, describing the impact of research engagement on chaplains themselves may encourage chaplains—many of whom may not have previously considered participating in research—to contemplate, initiate, or persevere in research endeavors. It may also encourage chaplains to obtain more formal research training and assert the value of research literacy for chaplains and chaplaincy students.
